# Predictors of e-cigarette usage among individuals with asthma and COPD

**DOI:** 10.1186/s12889-025-24084-2

**Published:** 2025-10-02

**Authors:** Tucker Rathe, Mario F. Perez, Felicia Tanu, Nkiruka C. Atuegwu, Eric M. Mortensen

**Affiliations:** 1 University of Connecticut School of Medicine, Farmington, CT 06032 USA; 2 VA North Texas Health Care System, Dallas, TX 75216 USA; 3 UConn Health, 263 Farmington Avenue, Farmington, CT 06030 USA

**Keywords:** E-cigarette, Asthma, COPD

## Abstract

**Background:**

Our study examined what demographic and health factors were associated with motivations for e-cigarette use in those with asthma and COPD.

**Methods:**

The analysis included participants ≥ 18 years old in Wave 5 of the Population Assessment of Tobacco and Health who reported e-cigarette use and had asthma or COPD. We used multivariable logistic regression models, adjusted for survey weights, to examine the associations of potential reasons for e-cigarette use, including affordability and attempts to minimize or quit cigarette smoking.

**Results:**

Seven hundred twenty-five participants (weighted *n* = 2,588,403) met the inclusion criteria. Factors associated with using e-cigarettes to help reduce cigarette use included being > 45 years old (odds ratio 1.9, 95% confidence interval 1.1–3.2) and having experienced wheezing in the past year (2.1, 1.3–3.4). Experiencing wheezing was also the only factor associated with using e-cigarettes to help quit smoking (2.6, 1.5–4.5). Being Hispanic and being moderately to very worried about the health impacts of any tobacco product usage, which includes electronic products, conferred a lower likelihood of using e-cigarettes to cut down on cigarettes (0.5, 0.3–0.98; 0.4, 0.3–0.8). Those who identified as male or being moderately to very worried about the health impacts of their tobacco product usage were significantly less likely to have initiated e-cigarettes for smoking cessation (0.6, 0.4–0.9; 0.3, 0.2–0.5).

**Conclusions:**

Factors associated with the uptake of e-cigarettes as a smoking cessation tool include age, gender, and ethnicity. Wheezing, as a symptom of respiratory illness, appears to have the strongest association with e-cigarette usage as a form of smoking cessation. At the same time, concern about the health impacts of tobacco products is the strongest negative predictor.

## Background

Cigarette smoking is the leading cause of preventable morbidity and mortality in the US and across the world [1]. There are over 6000 toxicants in cigarette smoke, and many of them are known to be human carcinogens that affect not only people who smoke cigarettes but also those around them [2]. E-cigarettes were introduced in 2007 and contain lower amounts of some toxicants compared to combustible tobacco and are promoted as a safer alternative to cigarettes [3] since the aerosol produced by e-cigarettes has lower toxicant levels than those found in cigarette smoke [4]. Furthermore, multiple studies have shown that nicotine-containing e-cigarettes may serve as a cessation tool with greater efficacy than current smoking cessation methods [5].

The premise that e-cigarettes are considered safer than cigarette smoke has enticed many individuals who smoke cigarettes to consider using e-cigarettes [6]. Moreover, e-cigarettes have on average become more affordable across all age groups and states in recent years [7], and are available in multiple attractive devices and flavors, which may attract youth who do not already smoke cigarettes to uptake their use [8].

Prior research has demonstrated associations between e-cigarette use and several adverse respiratory health outcomes, including asthma, chronic obstructive pulmonary disease (COPD), bronchitis, increased respiratory symptoms such as cough, and a potential increased risk of COVID-19 [9]. Research has indicated that e-cigarettes affect the respiratory system, and it has been shown that healthy individuals, after only five minutes of e-cigarette use (“vaping”), had a significant increase in airway resistance [10]. For individuals with respiratory pathologies like asthma and COPD whose baseline lung function is already impaired with dynamic or fixed obstruction, e-cigarettes have the potential to worsen pulmonary symptoms and worsen their already established respiratory disease, as at least one study has shown that adolescents with asthma who vape experience detrimental short-term effects [11]. Yet, some individuals with asthma showed a higher prevalence of e-cigarette usage and reported more positive opinions about e-cigarettes than those who did not have asthma [12]. Similarly, e-cigarette users have higher blood levels of YKL-40, a marker of asthma and COPD that has also been found elevated during acute exacerbations [13], and e-cigarettes have the potential to affect the immune response, which can contribute to and exacerbate lung inflammation, a key feature of COPD [14]. The body of research examining health effects of e-cigarettes is not homogenously negative, with some studies concluding the use of the product is minimal or even beneficial for COPD symptoms when used as a form of smoking cessation [15]. However, the conflicting nature of findings across the research landscape about the effects of e-cigarettes should inspire hesitancy regarding their use. Despite the potential for worsening respiratory disease, there is a noticeable lack of literature examining why individuals with asthma or COPD use e-cigarettes.

The goal of our study was to identify factors associated with the potential reasons for the use of e-cigarettes among individuals with respiratory diseases such as asthma and COPD. Our a priori hypotheses were that affordability would be associated with e-cigarette use in older and lower-income demographics, while concerns about the health impacts of tobacco products overall would be negatively associated with e-cigarette use in those attempting to cut down or eliminate the use of traditional cigarettes.

## Methods

We used data from Wave 5 of the adult Population Assessment of Tobacco and Health (PATH) survey public-use files for this study. The adult PATH survey is a longitudinal study analyzing tobacco use, attitudes, beliefs, and health of civilian non-institutionalized adults in the US population [16]. Data collection for Wave 5 occurred between December 1, 2018, and November 30, 2019, and was conducted via in-person interviews and audio self-interviews using a computer. Additional information about the PATH study is publicly available [16]. The UConn Health Institutional Review Board determined that this study fit the criteria for exempt research.

### Inclusion criteria

There were 34,309 adult (18 or older) respondents for Wave 5. Questionnaire respondents were obtained by voluntary participation via random selection from a nationwide list of addresses. Participants with a prior diagnosis of chronic obstructive pulmonary disorder (COPD) or asthma who had also previously used or currently use e-cigarettes (*n* = 725, weighted *n* = 2,588,403) were included in these analyses. There were no specific exclusion criteria once inclusion criteria were met. The weighted sample size was obtained by implementing indicated PATH survey weights in observation of the database’s nationally representative sampling technique. E-cigarette use was defined as current established, current experimental, recent former established, or non-current 30-day electronic nicotine product users. Participants classified as having asthma and/or COPD were new baseline adult respondents told by a medical professional they had asthma and/or COPD or continuing adult respondents told by a medical professional they had asthma and/or COPD in the past 12 months.

### Outcomes

As we wished to identify potential reasons for e-cigarette use in those with asthma and COPD, we utilized the following PATH questions: (1) use/used electronic nicotine products because they are/were affordable, (2) use/used electronic nicotine products as a way of cutting down on your cigarette smoking, and (3) using/use electronic nicotine products as a way to quit smoking cigarettes. Reasons (2) and (3) applied to a subset of the sample that indicated history of traditional cigarette smoking (*n* = 606).

### Data and definitions

Data was collected on age, race, gender, income, education level, marital status, educational level, medications for pulmonary and cardiovascular conditions, mental health and illness, problems with listening and attention, and history of tobacco, alcohol, marijuana, methamphetamine, cocaine, and non-prescribed drug use in Wave 5, with the full list of covariates included in Table 1. Participants’ ages were defined as either above or below 45 years old. Race was defined in three separate binary categories: White only, Black only, and Others. Ethnicity was described as either Hispanic or not Hispanic. Education level was defined as having less than a high school education or having at least a high school diploma or GED (General Equivalency Diploma). Income level was defined as less than $50,000 or more than $50,000 per year. Marital status was defined as married, widowed/divorced/separated, or never married. Externalizing and internalizing problems such as instances of feeling anxious, depressed, having problems with attention, and having problems with listening were defined as either within the last year or not within the last year. The extent to which a participant was worried about the health effects of tobacco and electronic nicotine products was defined as either not at all to a little worried or moderately to very worried.

**Table 1:** Baseline demographic characteristics among e-cigarette users with certain reasons for usage

	Ever e-cig users—use/used electronic nicotine products:		
	Because they were/are affordable; (*n* = 376) (%) (weighted *n* = 1,342,399)	As a way of cutting down on cigarettes; (*n* = 263) (%) (weighted *n* = 938,966)	As a way to quit smoking cigarettes; (*n* = 219) (%) (weighted *n* = 781,876)
Age group ^a^			
18–44 years old	47.4	42.0	44.5
45 years or older	52.6	58.0	55.5
Gender ^b^			
Male	32.0	30.1	27.2
Female	68.0	69.9	72.8
Race ^c^			
White	78.7	84.2	82.8
Black	11.4	8.4	9.6
Other	9.9	7.4	7.6
Ethnicity ^d^			
Hispanic	11.6	6.7	7.8
Education ^e^			
Did not complete high school	16.8	21.0	23.9
Completed high school	83.2	79.0	76.1
Income ^f^			
Under $50,000	74.3	80.3	79.4
Over $50,000	25.7	19.7	20.6
Used in the past 12 months			
Recreational drugs ^g^	29.6	28.4	29.3
Respiratory medication ^h^	54.5	44.7	58.1
Cardiovascular medication ^i^	37.7	37.0	35.8
Experienced in the past 12 months			
Bad asthma attack ^j^	16.5	19.0	15.3
Wheezing ^k^	61.9	74.8	76.8
Problems with anxiety ^l^	58.7	65.5	61.8
Problems with depression ^m^	54.5	58.8	56.5
Problems with attention ^n^	54.0	54.4	51.1
Problems with listening ^o^	48.4	50.3	45.6
Psychiatric diagnosis ^p^	6.3	5.6	6.1
Took prednisone nearly every day for a month or longer ^q^	4.5	5.4	6.0
Moderately to very worried about the impact of tobacco products on health ^r^	55.5	38.7	33.3
Wave 5 Adult Ever Cigarette Smoker ^s^	Yes—606	No—119	

PATH Questionnaire Items used to create table:

^a^ R05R_A_AGECAT6

^b^R05R_A_SEX

^c^R05R_A_RACECAT3

^d^R05R_A_HISP

^e^R05R_A_AM0018_V2

^f^R05R_A_AM0030

^g^R05_AX0089_12M_01/R05_AX0220_12M_01

^h^R05_AX0283, ^i^R05_AX0219

^i^R05_AX0219

^k^R05_AX0047_12M

^l^R05_AX0163

^m^R05_AX0161

^n^R05_AX0166

^o^R05_AX0167

^p^R05_AX0756_12M

^q^R05_AX0700

^r^R05_AX0105

^s^R05R_A_EVR_CIGS

### Statistical analyses

Binary logistic regression models were created to investigate the relationship between the factors of interest and why participants use e-cigarettes. We chose these variables from the questionnaire a priori as relevant confounders to the outcomes. We created separate models incorporating 20 covariates (listed in Table 1) and their association with the three separate outcomes of (1) use/used electronic nicotine products because they are/were affordable, (2) use/used electronic nicotine products as a way of cutting down on your cigarette smoking, and (3) using/use electronic nicotine products as a way to quit smoking cigarettes.

The PATH study design utilized replicate weights and balanced repeated replication methods. Statistical analyses were performed using Stata version 18.0. Statistical significance was determined via a two-tailed *p*-value of ≤ 0.05. The PATH survey is subject to extensive quality control and standardization of collection of nationally representative data as directed by the National Institutes of Health and the Federal Drug Administration; as such, the authors did not elect to conduct sensitivity analyses.

## Results

The cohort included 725 participants (weighted *n* = 2,588,403). The cohort was similar between those 18–44 years old and those 45 years or older within all three outcome variable groups, while the female-to-male ratio among the cohort was approximately 2:1 overall. Most of the cohort completed high school, and most reported an income of under $50,000 a year. Further demographic information is listed in Table 1.

### Binary logistic regression models

**Fig. 1:**
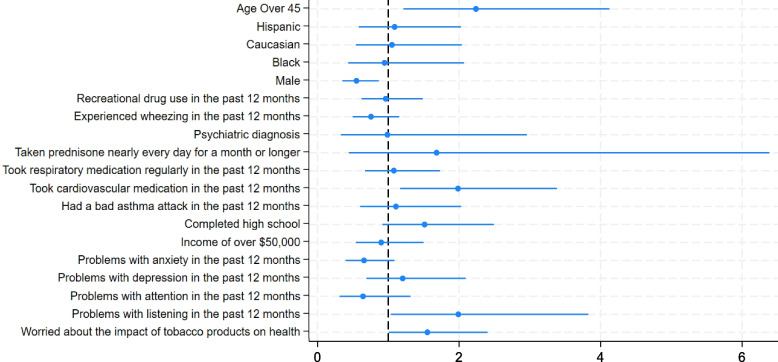
Associations between demographic and health status information and using e-cigarettes because they were affordable

The results of the binary logistic regression models are shown in Fig. 1. Respondents who were over 45 years old (odds ratio 2.24, 95% confidence interval: 1.22–4.12), those who were taking heart or blood pressure medication regularly (OR 1.99, 95% CI: 1.17–3.38), those who were moderately to very worried about the negative health effects of tobacco product use (OR 1.55, 95% CI: 1.00–2.40), and those who had experienced problems with listening to instructions within the past year (OR 1.99, 95% CI: 1.04–3.82) were more likely to have used e-cigarettes because they were affordable. Those who identified as male (OR 0.55, 95% CI: 0.35–0.87) were less likely to have used e-cigarettes because they were affordable (Fig. 1).

**Fig. 2:**
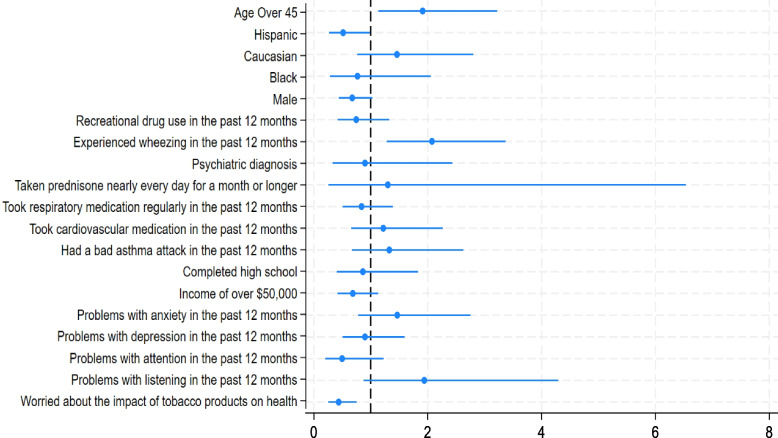
Associations between demographic and health status information and using e-cigarettes to cut down cigarette smoking

Respondents who were over 45 years old (OR 1.91, 95% CI: 1.13–3.22) and those who had experienced wheezing in the past year (OR 2.07, 95% CI: 1.28–3.36) were more likely to have used e-cigarettes to cut down on their cigarette smoking. Those who identified as Hispanic (OR 0.51, 95% CI: 0.27–0.98) and those who were moderately to very worried about the negative health effects of tobacco product use (OR 0.44, 95% CI: 0.25–0.75) were less likely to have used e-cigarettes to cut down on their cigarette smoking (Fig. 2).

**Fig. 3:**
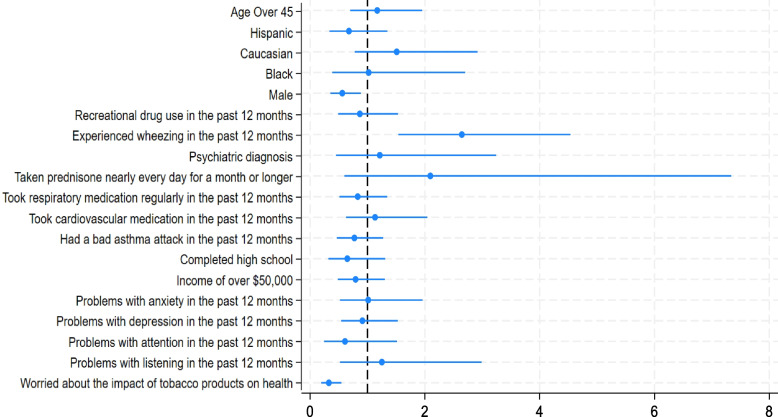
Associations between demographic and health status information and using e-cigarettes to quit smoking cigarettes

Respondents who had experienced wheezing in the past year (OR 2.64, 95% CI: 1.54–4.54) were more likely to have used e-cigarettes to help them quit smoking completely. Those who identified as male (OR 0.56, 95% CI: 0.36–0.88) and those who were moderately to very worried about the negative health effects of tobacco product usage (OR 0.33, 95% CI: 0.20–0.54) were less likely to have used e-cigarettes help them quit smoking completely (Fig. 3).

## Discussion

This study analyzed a nationally representative sample of individuals who currently use or had previously used e-cigarettes and have been diagnosed with either asthma or COPD. We found that age, gender, and ethnicity played statistically significant roles in the decision to use e-cigarettes as a way of decreasing usage of or quitting smoking. The strongest positive predictor of e-cigarette usage as a form of smoking cessation was experiencing wheezing. In contrast, the strongest negative predictor was being worried about the overall impact of tobacco products on health. E-cigarette usage due to affordability was most strongly associated with older age, concurrent prescription of heart or blood pressure medication use, and problems with listening and attention.

We found that using e-cigarettes because they were affordable was associated with being worried about the negative effects of tobacco product usage and problems with listening to instructions in the past year. The reasons behind these associations are unclear. Potentially, listening difficulties may coexist with more impulsive decision making, and affordable e-cigarette prices seen in stores being enough to warrant initiation without consideration of potential health effects. Additionally, listening difficulties may result in a higher frequency of educational and vocational tribulations, which may lead to e-cigarette use as a form of stress relief, a possibility that informed the decision to include it as a covariate in this analysis. However, this association was stronger among those over 45 years old and those taking heart or blood pressure medication regularly. These stronger associations may be due to the prevalent notion of e-cigarettes being a nicotine delivery option that mitigates health risks, but the risks of e-cigarettes themselves should not be dismissed as a result. While some studies have indicated that using e-cigarettes was correlated with better blood pressure control [17], that is only in comparison to those who smoked traditional cigarettes, not in comparison to those who used neither product. Furthermore, e-cigarette usage itself has been shown to promote vascular remodeling and increased sympathetic nervous system activation, more likely leading to exacerbation rather than mitigation of cardiovascular conditions [18]. It should also be noted that although e-cigarettes may be perceived as affordable by some subjects, the total healthcare and hospital costs in the US attributed to e-cigarettes have already surpassed other forms of nicotine delivery, including cigars and smokeless tobacco products that have been in the market for far longer [19]. This would make e-cigarette use a less financially favorable tobacco alternative in the long term, especially for individuals with asthma and COPD who already incur high healthcare costs and frequent emergency department utilization from episodic exacerbations of these conditions [20].

Using e-cigarettes either to cut down on cigarette smoking or to quit smoking entirely was somewhat associated with being over 45 years of age, male gender, and Hispanic ethnicity. Still, it was most strongly associated with experiencing wheezing. Worrying about the negative effects of tobacco product usage exhibited a strong negative association. One potential explanation for the strong association with wheezing could be that these subjects have more severe forms of COPD or asthma and that their respiratory disease is less controlled with worsening quality of life as a result of cigarette smoking. If an individual who currently smokes cigarettes is looking to decrease or eliminate their cigarette usage and has also experienced symptomatic manifestations of their asthma or COPD, they may view non-combustible options like e-cigarettes as the more acceptable option, an alternative that would not impact their lung conditions as severely. However, there is a growing body of scientific evidence that rebuts that presumption [21]. Additionally, e-cigarettes can also exhibit effects beyond the respiratory system. Recent research via the American College of Cardiology demonstrated that those who used e-cigarettes at any point in their lives are 19% more likely to develop heart failure compared to those who had never used the product [22].

Our findings could serve as a foundation to further research why these factors were such strong predictors and to inform policy initiatives that increase awareness as to the potential harms of e-cigarettes, which may help to decrease the prevalence of their usage. For example, if a patient who smokes and has asthma or COPD is reporting bothersome wheezing to his physician at an annual physical, it may be particularly important for that physician dedicate time to establishing a smoking cessation plan, as that patient may be more likely to initiate e-cigarette usage. The results of this analysis suggest that the relative novelty of the product may be its most polarizing feature, as the scientific community’s inability to provide concrete evidence as to its long-term harms at present may be a significant selling point. To others, the fear of the unknown is as strong a deterrent as any other. Therefore, the most effective messaging strategy for e-cigarette use might be one that involves communicating the uncertainty of the product’s safety profile and urging caution, keeping those who had not used tobacco products previously from initiating and steering individuals who smoke cigarettes towards FDA-approved strategies including nicotine replacement therapies and pharmacological approaches, which demonstrate little to no risk of exacerbating pulmonary disease [23].

There has been evidence demonstrated in past studies that e-cigarettes have conferred a higher smoking cessation success rate when compared to nicotine replacement therapy [24]. In the grand scheme of the advancement of public health, complete cessation of the use of combustible tobacco products is the goal, and no matter the cessation method, there is a significant reduction in harm when an individual stops using traditional cigarettes. Recent data suggests that approximately 1 in 7 adults who currently smoke live with some degree of disability, with the most prevalent deficits being related to mobility and cognition [25]. In that way, the best method is whichever method will be best adhered to, especially considering environmental and cost-related factors. However, ensuring awareness of nicotine replacement therapy and its widespread availability to those who will utilize the healthcare system for smoking cessation counseling is necessary, not only for the notable absence of possibility of pulmonary insult, but also other components, including financial considerations, as nicotine replacement therapy is often covered by insurance [26]. For patients who still indicate a preference for e-cigarettes, providing evidence-based counseling as to the potential benefits and harms ensures that their ultimate decision is an informed one. For those with asthma and COPD, nicotine replacement therapy offers a path to smoking cessation without pulmonary involvement, but e-cigarettes also provide a path that has shown to be effective in helping individuals who smoke cigarettes quit. As shown in this study, certain characteristics may push specific demographics towards the e-cigarette method, while some may be pushed away from that strategy. Discovering which philosophy will lead to the quickest, most assured cessation of cigarette use for a particular individual while minimizing unintended consequences, whether they be personal, financial, or health-related, should be the aim of public health interventions.

The nationally representative nature of the PATH database confers high statistical power and a corresponding higher confidence that these findings are applicable on a national scale. Additionally, the substantial number of questions asked of participants within the questionnaire allowed for a robust and thorough study of e-cigarette attitudes from multiple vantage points. Finally, this study utilized Wave 5 of the PATH, the latest collected dataset from before the COVID-19 pandemic. This allowed us to assess up-to-date perspectives on e-cigarettes without the confounding impact the pandemic and subsequent quarantine period may have had on consumer beliefs and behaviors.

Our work, however, is not without limitations. The female-to-male ratio of the sample is 2:1, and Hispanic respondents made up only a limited percentage, which may limit generalizability, especially with respect to the statistically significant finding of Hispanic respondents being less likely to use e-cigarettes to decrease traditional cigarette smoking. Though the weighted sample size is 2,588,403, the unweighted sample size of 725 may confer less power in subgroup analysis.

The data is subject to recall bias. In some cases, the participant may have had to think years back about what drove them to buy and use e-cigarettes. If their beliefs about e-cigarettes have changed since they first used the product, they may only endorse their current point of view in their survey response rather than incorporating the aspects of the product that led them to use it before. Further, self-reported data is subject to bias in that there is nothing to guarantee respondent candor regarding habits or motivations even in instances where they do accurately remember, though the anonymous nature of the collected data may help to reduce social desirability bias. The PATH database does not account for confounding like smoking cessation programs, e-cigarette advertisement density, or others that may only affect certain individual or subgroups of respondents’ behaviors rather than the entire sample. Finally, as a cross-sectional study evaluating a single wave of the PATH data, causation cannot be determined, only correlation. Subsequent waves of PATH data to be analyzed in a longitudinal fashion for future studies would be illuminating in evaluating if e-cigarette usage has persisted and if motivations for use are unchanged for specific participants, and public health recommendations made in response to this type of study would have more robust support than can be offered in this cross-sectional analysis. Future studies may also benefit from the inclusion of pack-years, cessation attempts, and quantitative and qualitative metrics of nicotine dependence in the analysis to discern if more advanced tobacco usage has significant effects of motivations for initiation.

## Conclusions

In conclusion, we found that the reasons people with asthma or COPD choose to use e-cigarettes vary between subgroups. Still, the most significant associations were a higher likelihood among those with smoking history of using e-cigarettes for smoking cessation if they had wheezed in the past year and a lower likelihood if they were concerned about the health effects of tobacco products overall. The findings of this study indicate a preliminary direction of intervention that may decrease overall tobacco product usage. Future research as to the long-term health effects of e-cigarettes, specifically related to the harm reduction they may or may not afford in comparison to traditional cigarettes for those with pulmonary disease, is needed so we can better counsel the public as to the potential dangers of these products.

## Data Availability

The datasets analyzed during the current study are available in the Population Assessment of Tobacco and Health repository, https://www.icpsr.umich.edu/web/NAHDAP/studies/36498/datadocumentation.
